# Bioinspired Structural Composite Flexible Material with High Cushion Performance

**DOI:** 10.1002/advs.202304947

**Published:** 2023-12-03

**Authors:** Zhiqiang Zhuang, Zhihui Qian, Xu Wang, Xiaolin Xu, Boya Chen, Guangsheng Song, Xiangyu Liu, Lei Ren, Luquan Ren

**Affiliations:** ^1^ Key Laboratory of Bionic Engineering Ministry of Education Jilin University Changchun 130022 China; ^2^ Institute of Structured and Architected Materials Liaoning Academy of Materials Shenyang 110167 China; ^3^ Orthopaedic Medical Center The Second Hospital of Jilin University Changchun 130022 China; ^4^ Department of Mechanical Aerospace and Civil Engineering University of Manchester Manchester M13 9PL United Kingdom

**Keywords:** cushioning performance, impact force, integrated bionic strategy, structural composite flexible materials

## Abstract

Impacts occur everywhere, and they pose a serious threat to human health and production safety. Flexible materials with efficient cushioning and energy absorption are ideal candidates to provide protection from impacts. Despite the high demand, the cushioning capacity of protective materials is still limited. In this study, an integrated bionic strategy is proposed, and a bioinspired structural composite material with highly cushioning performance is developed on the basis of this strategy. The results demonstrated that the integrated bionic material, an S‐spider web‐foam, has excellent energy storage and dissipation as well as cushioning performance. Under impact loading, S‐spider web‐foam can reduce peak impact forces by a factor of 3.5 times better than silicone foam, achieving unprecedented cushioning performance. The results of this study deepen the understanding of flexible cushioning materials and may provide new strategies and inspiration for the preparation of high‐performance flexible cushioning materials.

## Graphical abstract

An integrated bionic strategy is proposed, and a bioinspired structural composite material with highly efficient cushioning performance is designed on the basis of this strategy. The integrated bionic material, an S‐spider web‐foam, achieves unprecedented cushioning performance. The results of this study may provide new strategies and inspiration for the preparation of high‐performance flexible cushioning materials.

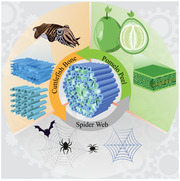

## 1 Introduction

Impacts are ubiquitous and often bring loss of life and equipment failure, therefore, the development of efficient cushioning materials for protection from impacts has attracted great attention for years.^[^ ^1^ ^]^ Many cushion materials use inelastic mechanisms to dissipate impact energy, such as plastic deformation,^[^ ^2^ ^]^ fracture,^[^ ^3^ ^]^ and unstable elastic buckling.^[^ ^4^ ^]^ However, this approach is mostly at the expense of its structure, and these protective materials cannot be reused. Flexible materials with cushioning properties offer the most effective way of reducing peak impact forces, which has led to great interest in exploring flexible cushioning materials.

In order to reduce the risk of damage to human body and destruction of industrial products by impact loads, flexible materials such as liquid crystal elastomers,^[^ ^5^, ^6^ ^]^ gels,^[^ ^7^ ^]^ non‐Newtonian fluids^[^ ^8^, ^9^ ^]^ and foams^[^ ^10^, ^11^ ^]^ have been well developed and widely used for their excellent cushioning properties. Polyethylene, polypropylene, polyvinyl chloride, polystyrene and polyurethane foam are more commonly used flexible cushioning materials in human daily life.^[^ ^12^, ^13^, ^14^ ^]^ Under impact load, these polymer foams absorb energy due to compaction and limit the transfer of load to the protected object, and it is widely used in the field of product protection.^[^ ^15^ ^]^ However, polyethylene and polystyrene foams are flammable, ignitable and release toxic gases, and polystyrene foams have limited loading capacity.^[^ ^16^ ^]^ Polypropylene foam is susceptible to oxidation, and to be decomposed by ultraviolet light.^[^ ^17^, ^18^, ^19^ ^]^ Single polyurethane foam has limited energy absorption capacity which results in weak cushioning performance and cannot offer enough protection for products.^[^ ^20^ ^]^ However, filling these porous materials with non‐Newtonian fluids or shear‐thickening gels is an effective way to improve their cushioning properties, e.g., PU foam with non‐Newtonian fluids reduces the impact force by a factor of 2 compared to PU foam.^[^ ^21^ ^]^ Since the modulus and viscosity of shear thickening gel (STG) increases with strain rate, it has good energy absorption capacity under impact loads.^[^ ^22^ ^]^ The composites formed by combining STG with porous structure such as ethylene vinyl acetate foam,^[^ ^23^ ^]^ high‐performance fibres,^[^ ^22^ ^]^ multilayer ultra‐high molecular weight polyethylene (UHMWPE),^[^ ^24^ ^]^ and SiO_2_ particle,^[^ ^25^ ^]^ respectively, have good energy‐absorbing properties, and the cushioning performance is improved to varying degrees compared with their respective base materials. The coupling of STG and UHMWPE can reduce the impact force by up to 50%.^[^ ^24^ ^]^ In addition, liquid crystal elastomers (LCEs) are different from conventional foams and STG due to their multilevel energy dissipation mechanisms, and their extraordinary energy dissipation capabilities have been at the forefront of recent polymer research.^[^ ^6^, ^26^ ^]^ Upon deformation, the rotation of mesogen provides a second mechanism to dissipate energy in addition to the viscoelastic relaxation of polymer chains, which enables LCEs to enhance the energy dissipation and reduce an impact force by a factor of 2.6 times more than non‐LCE foams.^[^ ^26^ ^]^ These studies have provided with a comprehensive understanding of the cushioning properties as well as the energy absorption strategies of existing flexible cushioning materials. These strategies, through material modification or the incorporation of additional energy‐absorbing mediums in conventional foams, can improve the cushioning performance of the materials. However, the cushioning performance of these reported flexible materials still lags behind that of the well‐known energy‐absorbing structural materials in nature, e.g., the S‐shaped structure of the cuttlefish bone increases energy absorption by 60% compared to the contrasting structures,^[^ ^27^ ^]^ spider web dissipates up to 70% of dynamic impact energy,^[^ ^28^ ^]^ and pomelo peel allows the fruit to endure thousands of newtons of decelerating force.^[^ ^29^ ^]^

Based on nature inspirations, in this study, we proposed an integrated bionic design strategy to develop a bioinspired structural composite material with high cushioning performance. The developed structural composite flexible material has a wide range of potential applications for the protection of electronic devices, packaging and transport, shoe insoles and protection of industrial equipment, providing a new approach to the design and preparation of materials with excellent energy absorption and cushioning performance.

## 2 Results and Discussion

### 2.1 Design of Integrated Biomimetic Materials and Characterization of Bio‐Prototypes

**Figure 1:**
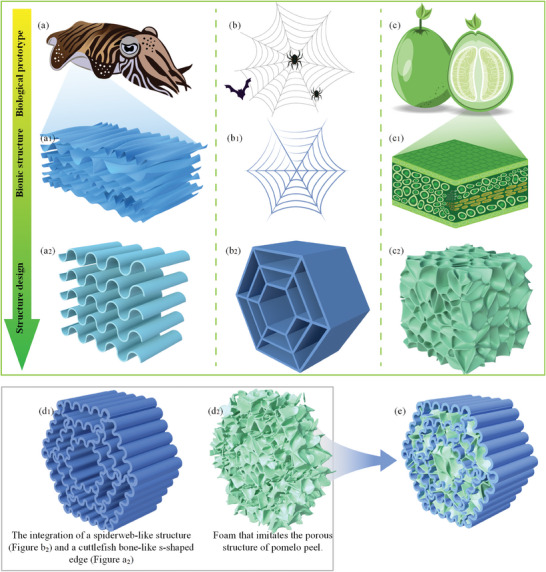
Biological prototypes and design strategies for integrated bionic design in this study. a) Cuttlefish, the first biological prototypes used for integrated bionic design. a_1_) Schematic diagram of the S‐shaped structure of cuttlefish bone. a_2_) Bionic design of the S‐shaped structure of cuttlefish bone. b) Spider web, the second biological prototype used for integrated bionic design. b_1_) Schematic diagram of the structure of spider web. b_2_) Bionic structure of spider web. c) Pomelo, the third biological prototype used for integrated bionic design. c_1_) Schematic diagram of the porous structure of pomelo peel. c_2_) Bionic design of the porous structure of pomelo peel. d_1_) The integration of a spiderweb‐like structure (Figure b_2_) and a cuttlefish bone‐like s‐shaped edge (Figure a_2_). d_2_) Foam that imitates the porous structure of pomelo peel. e) The S‐shaped spider web integrated with porous foam to form a flexible composite of porous materials with an S‐shaped spider web structure that was designed in this study.

**Figure 2:**
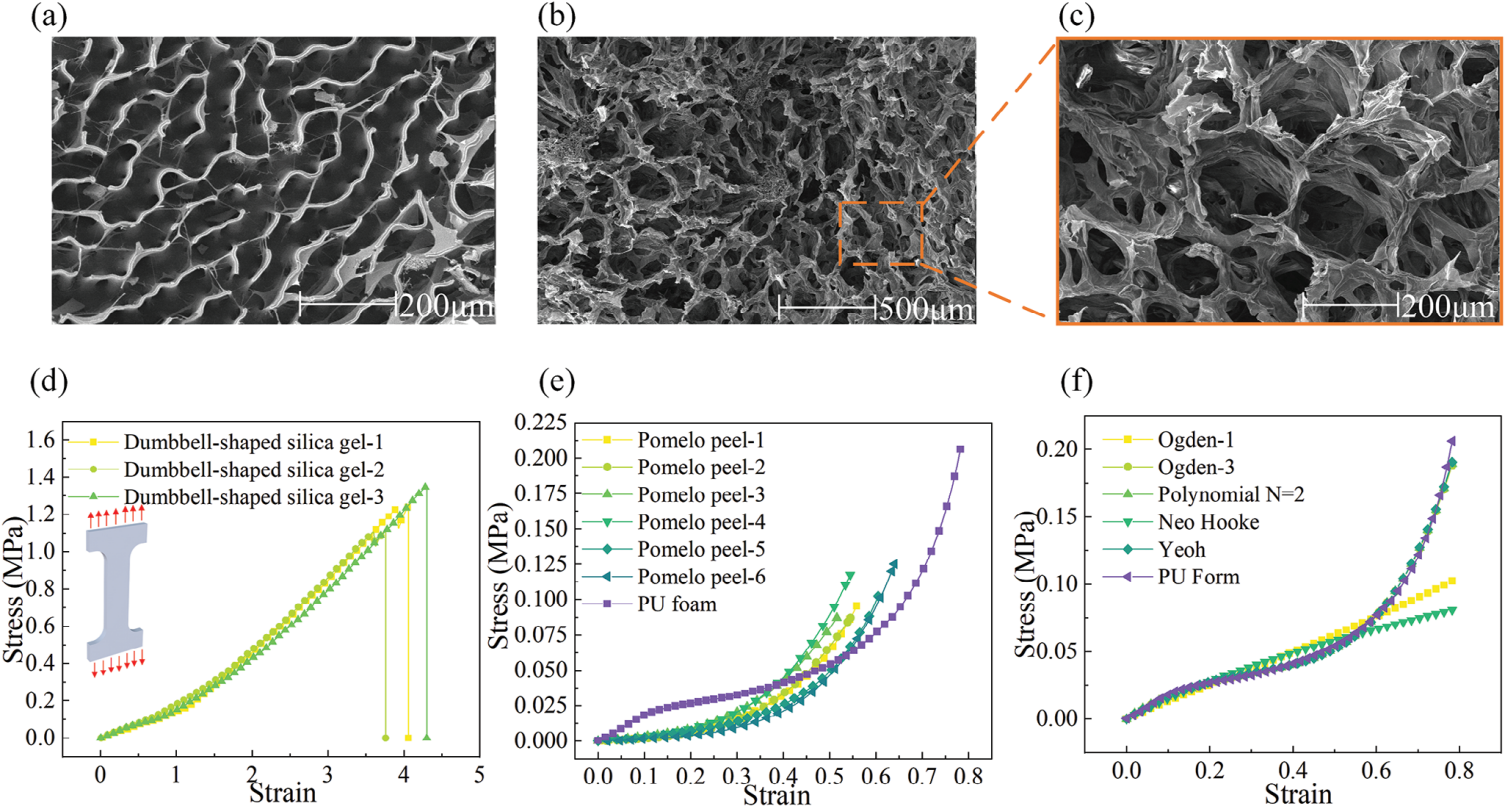
Structural characterization and mechanical property tests of the bio‐prototypes. a) Image of the S‐shaped structure of cuttlefish bone observed by SEM. b) and c) Images of the porous structure of pomelo peel observed by SEM. d) Test results for dumbbell‐shaped specimens of silicone under tensile loading. e) Stress‒strain curves of pomelo peel and PU foam under compressive loading. f) Results of fitting with several mathematical hyper‐elasticity models for PU foams for finite element simulations, with the specific parameters shown in the Supporting Information.

Nature provides a natural template for innovative design, and integrated bionics provides many new ideas for the development of multifunctional materials. Examples range from cobweb structures that dissipate up to 70% of dynamic impact energy in air^[^ ^28^, ^30^ ^]^ to the S‐shaped structure of cuttlefish bones with efficient energy absorption in the deep sea.^[^ ^31^, ^32^, ^33^ ^]^ In the plant world, the porous structure of the pomelo peel dissipates up to 98 J of energy from an impact and has excellent cushioning properties to effectively protect the pomelo fruit.^[^ ^29^ ^]^ Inspired by the S‐shaped structure of cuttlefish bone, spider web structures and the porous material in pomelo peel, we integrated all three structural materials into one bionic design. The design concept is shown in **Figure** **1**. First, we characterized the structure of cuttlefish bone, as previously reported in the literature,^[^ ^31^ ^]^ and we found that this bone had a curved S‐shaped structure, as shown in **Figure** **2**a. We used it as one of the biological prototypes in this study, as shown in Figure 1 a_2_. According to previous studies, spider webs have polygonal web structures.^[^ ^34^ ^]^ We considered a regular hexagonal web structure and used it as the second bionic structure in this study, as shown in Figure 1 b_2._ Pomelo peel was observed by scanning electron microscope (SEM) (Zeiss Sigma‐300, Germany). We found a porous structure as reported previously and shown in Figure 2b. Figure 2c is an enlarged view of Figure 2b, which more clearly characterized the internal structure of the pomelo peel. This porous structure was employed as one of the bionic structures in the study, as shown in Figure 1 c_2_. In this study, a spider web was designed by imitating a hexagonal web structure, as shown in Figure 1 b_2_. We designed a spider web where each side had an S‐shape based on the structure of the cuttlefish bone, thus forming an S‐shaped spider web, as shown in Figure 1d. There were cavities between the S‐shaped spider webs, and each cavity was filled with porous, soft polyurethane (PU) foam that was inspired by the porous structure of the pomelo peel. This was the final design of the bioinspired S‐shaped spider web multilayer composite prepared in this study.

**Figure 3:**
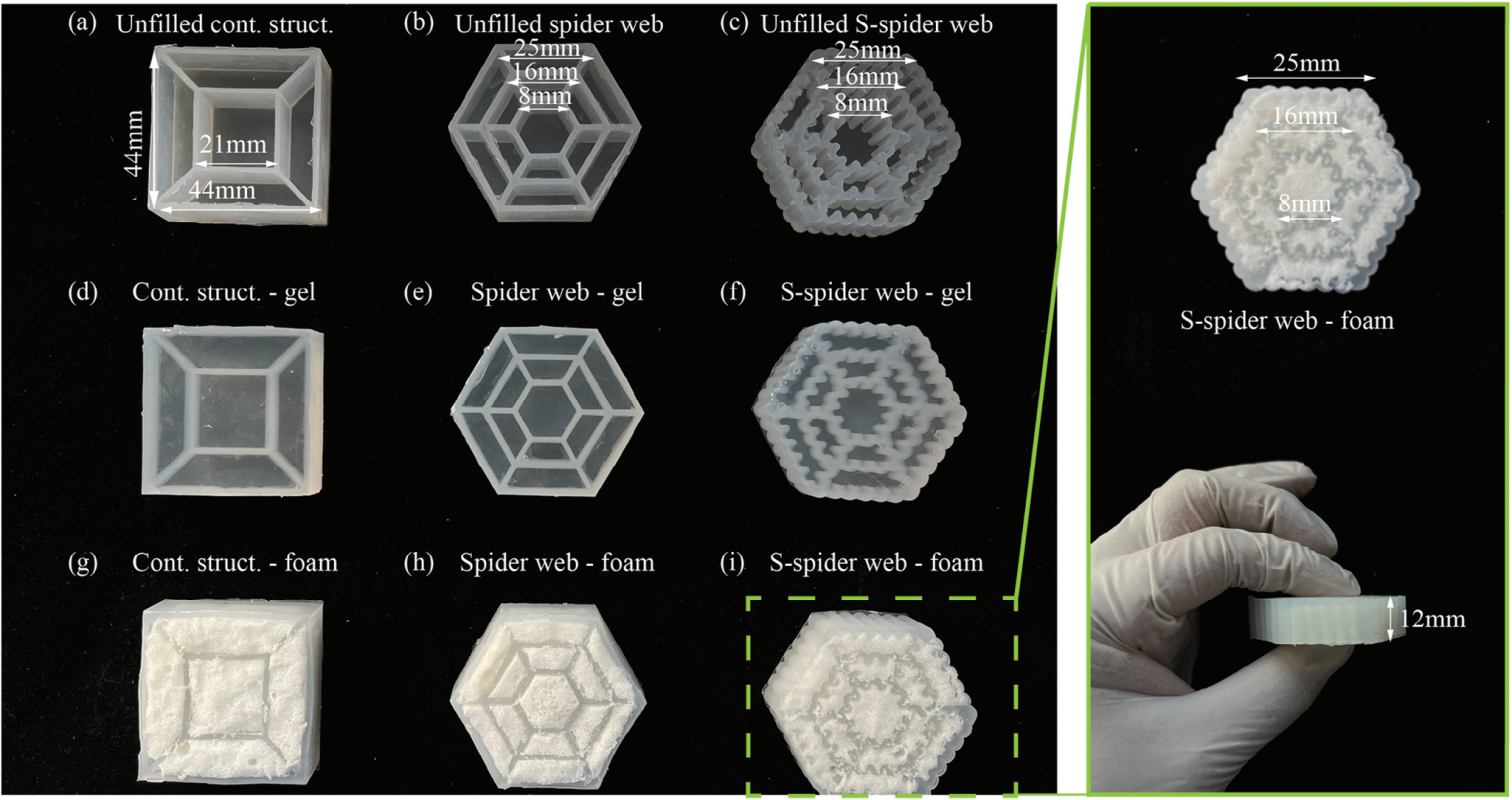
Bioinspired structural composite materials. a) Unfilled contrast structure. b) Unfilled spider web structure. c) Unfilled s‐type spider web structure. d) Contrast structure filled with Ecoflex Gel. e) Spider web structure filled with Ecoflex Gel. f) S‐type spider web structure filled with Ecoflex Gel. g) Contrast structure filled with PU foam. h) Spider web structure filled with PU foam. i) S‐type spider web structure filled with PU foam.

The S‐shaped spider web was prepared by casting silicone, the details of the preparation process are described in the Experimental Section. We chose silicone with hardness of 16A, which is a common hardness value of some cushioning materials.^[^ ^35^, ^36^ ^]^ Silicone was prepared in a dumbbell‐shaped specimen and tested to characterize its mechanical properties. The stress‒strain curves under tensile loading showed insignificant nonlinearity, as in Figure 2d. The mechanical characteristics of this variable stiffness were very similar to those of spider webs.^[^ ^37^ ^]^ The PU foam was prepared as rectangular specimens, as shown in the Supporting Information, Figure 2. Its stress‒strain curves under compressive load showed obvious nonlinear mechanical behavior, as in Figure 2e, which was very close to the mechanical properties of pomelo peel. PU foam was used to fill each cavity in the S‐shaped spider web. The dimensions of the S‐shaped spider web structure are shown in **Figure** **3**i. The overall thickness of the integrated bionic material was 12 mm. The integrated bionic material was a three‐level web; the side lengths of the first‐, second‐ and third‐level webs were 8, 16 and 25 mm, respectively. The S‐shaped side had the shape of a regular wave, the side width was 1.5 mm, and the amplitude was 1 mm, as shown in Figure 3 in the Supporting Information. In order to make comparison with integrated bionic structure, contrasting structures was employed in this study. The contrasting structure either have no spider web structure or no S‐shaped edge imitating a cuttlefish bone. There were three cases for the filling inside the web cavities: PU foam, Ecoflex Gel (Gel), which was soft and a good cushion (Smoothon, PA, USA), and no filling at all. Therefore, nine different structural composite materials were prepared, all with the same volume, as shown in Figure 3. Figure 3a,d and g show the materials that have neither spider web structure nor S‐shaped edge imitating cuttlefish bone in their structure, with no filling, Gel, and PU foam inside their cavities, respectively. Figure 3b,e,h indicate the materials that have spider web structure, without S‐shaped edge, with no filling, Gel, and PU foam inside their cavities, respectively. Figure 3c,f,i show the materials that have both spider web structures and S‐shaped edges, with no filling, Gel, and PU foam inside their cavities, respectively.

### 2.2 Mechanical Characterization of Integrated Bionic Materials

**Figure 4:**
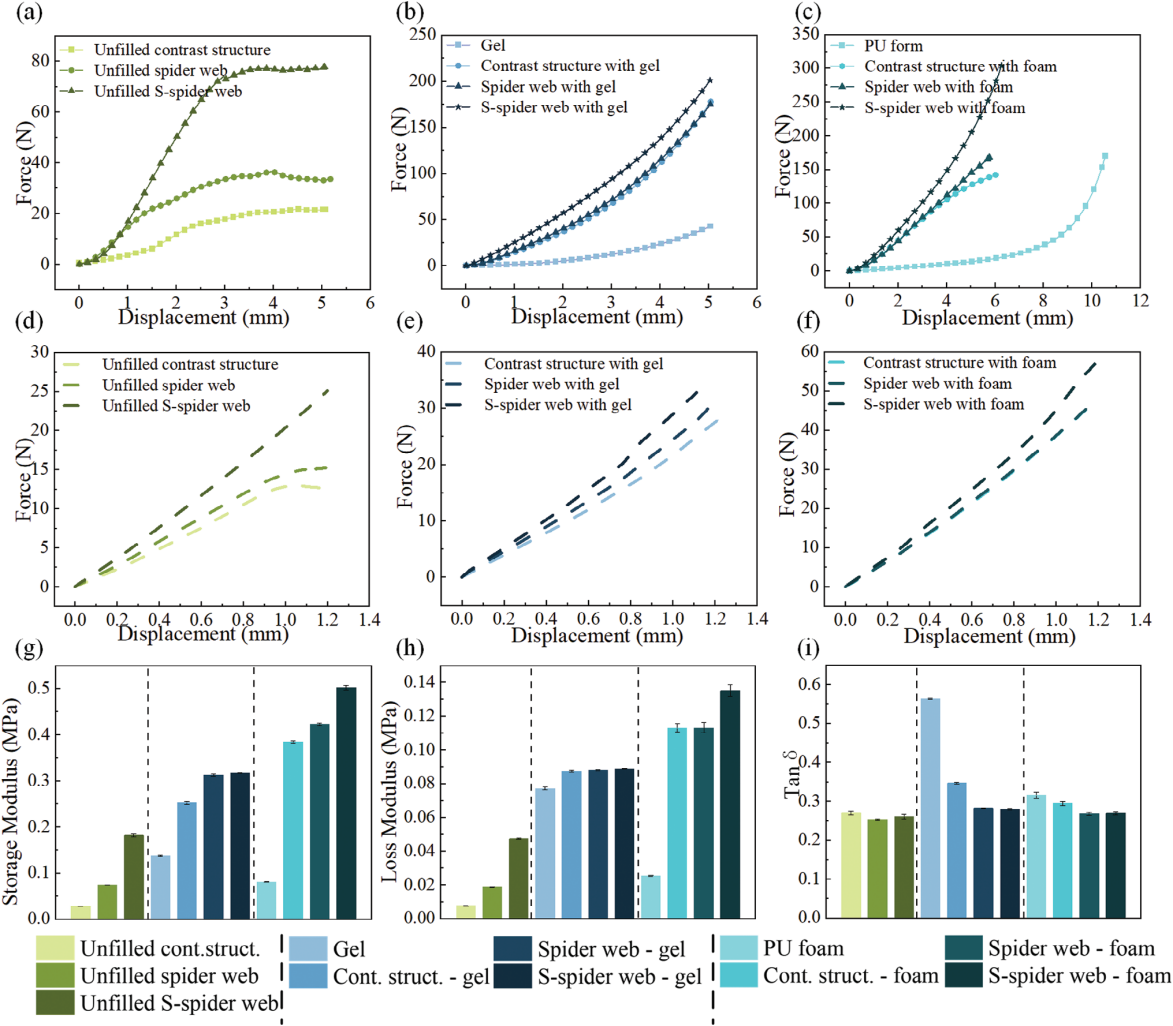
Mechanical property tests of bioinspired structural composite materials. a) Load‒displacement curves of three structural composite materials without filling under compression load. b) Load‒displacement curves of three structural composite materials filled with Gel. c) Load‒displacement curves of three structural composite materials filled with PU foam. d) FE simulation results for three structural composite materials without filling. e) FE simulation results for three structural composite materials filled with Gel. f) FE simulation results for three structural composite materials filled with PU foam. g) Storage modulus of eleven different structural composite materials. h) Loss modulus of eleven different structural composite materials. i) Values of the loss factor (Tanδ) of eleven different structural composite materials.

Nine integrated structural composite materials, Gel, and PU foam were tested under compressive loads using a universal testing machine (WDW‐2000, Wenzhou Weidu Electronics Co., China), and the test results are shown in **Figure** **4**a–c. Abaqus (Simulia, Providence, USA) is used to perform FE simulation of the bioinspired structural composite materials. Based on the results of fitting the mathematical model of hyperelasticity to PU foam, silicone and Gel, as shown in Figure 2f and Supporting Information Figure 1a, the PU foams uses the Yeoh hyper‐elasticity parameters, Gel and silicone use the Ogden‐3 hyper‐elasticity parameters respectively. The dashed lines in Figure 4d,e,f show the results of simulations corresponding to the test; the details of the simulation are in the Supporting Information. All materials exhibited significant nonlinear mechanical behavior under compressive loading.

**Figure 5:**
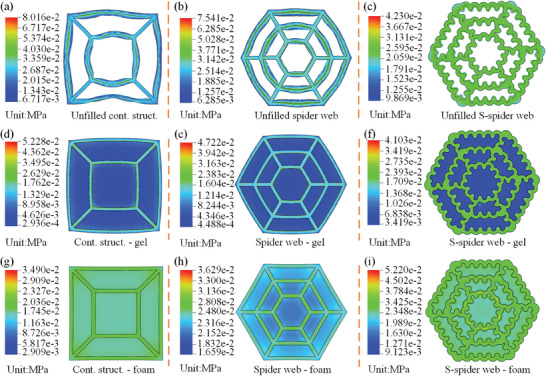
FE results for bioinspired structural composite materials. a) FE simulation results of an unfilled contrast structure. b) FE simulation results of an unfilled spider web. c) FE simulation results of an unfilled S‐spider web. d) FE simulation results of a contrast structure‐gel. e) FE simulation results of a spider web‐gel. f) FE simulation results of an S‐spider web‐gel. g) FE simulation results of a contrast structure‐foam. h) FE simulation results of a spider web‐foam. i) FE simulation results of an S‐spider web‐foam.

When there was no filling inside the web cavity, the spider web structure material with S‐edges that imitated cuttlefish bone (S‐spider web) was stiffer than the contrast structure, which was consistent with the prediction of the FE results. These structures undergo buckling under compressive loads, and the S‐spider web structure has a higher stiffness than the other structures, perhaps due to the S‐shaped structure of the edges, which makes it difficult to undergo buckling deformation, as shown in Figure 4d and **Figure** **5**a–c. The results show that at the same compressive strain, the bioinspired structure absorbs more energy due to the S‐shaped edges, which results in better cushioning performance, as shown in Figure 4a,d and Figure 5a,b,c. The S‐spider web‐Gel was the stiffest compared to other structures when the web cavity was filled with Gel, i.e., it requires the highest force for the same deformation, and therefore it has the best energy absorption compared to the other structures, as shown in Figure 4b,e and Figure 5d–f. The FE simulation results show that the coupling of the S‐spider web structure with the Gel results in a better energy‐absorbing property of the S‐spider web‐Gel, in which the S‐spider web structure contributes the most, as the stresses are mainly concentrated in its cross‐section, as shown in Figure 5f. When the web cavity was filled with PU foam, the stiffness of the S‐spider web‐form was still greater than that of the PU foam, and this result was consistent with the FE simulation. This suggests that the coupling of the S‐spider web structure with the PU foam results in excellent energy absorption properties of the S‐spider web‐foam, as shown in Figure 4c,f and Figure 5g–i. When subjected to compressive load, the coupling of the S‐spider web structure and the PU foam makes the S‐spider web‐form absorb more energy, in which the S‐spider web structure and the PU foam contribute as much as the S‐spider web structure, as the stress is uniformly distributed in the cross‐section of the S‐spider web‐form, as shown in Figure 5i.

The dynamic mechanical properties of nine structural composite materials, Gel and PU foam were measured by using a TA Electroforce‐3100 (Waters Technology Co. Ltd., USA). Displacement control was employed, the dynamic amplitude of the test was 1 mm, and the frequency was 1 Hz. The storage modulus, loss modulus, and loss factor (Tanδ) of the material were obtained based on the test. The storage modulus is the amount of energy stored due to elastic deformation when material deformation occurs, which reflects the amount of material elasticity. The loss modulus, also called the viscous modulus, is the amount of energy loss due to viscous deformation, which reflects the viscosity of the material.^[^ ^38^, ^39^ ^]^ The loss factor Tanδ is the ratio of the loss modulus and storage modulus, which reflects the viscoelasticity of the material.^[^ ^38^ ^]^ When the same filling was inside the web cavity, all the structural composite materials with the S‐spider web had larger storage and loss modulus, as shown in Figure 4g,h. For the unfilled S‐spider web, the storage modulus was larger by a factor of 6.4, and the loss modulus was larger by a factor of 6.2, compared to the unfilled contrast structure. The storage modulus of the S‐spider web‐Gel was 2.32 times higher than that of the Gel, and the loss modulus was 1.15 times higher than that of the Gel, when the filling inside the web cavity was Gel. When the filling inside the web cavity was PU foam, the storage modulus of S‐spider web‐foam was 6.2 times that of PU foam, and the loss modulus was 5.3 times that of PU foam. Therefore, the S‐spider web‐foam had good storage properties and damping, which is important for the absorption and dissipation of impact energy. The loss factors (Tanδ values) of the nine integrated structural composite materials as well as the Gel and PU foam were much less than 1; all of the materials were solids with viscoelastic material properties, as shown in Figure 4i.

### 2.3 Characterization of the Cushioning Properties of the Integrated Bionic Materials

**Figure 6:**
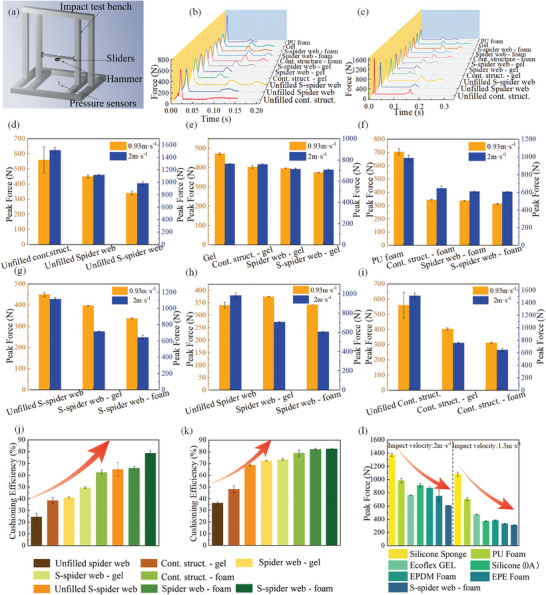
Cushioning performance test results of bioinspired structural composite materials. a) The cushioning test rig built in this laboratory and used in this study. b) Impact test results for nine structural composite materials at a dropped hammer impact velocity of 0.93 m s^−1^. c) Impact test results for nine structural composite materials at a dropped hammer impact velocity of 2 m s^−1^. d) Peak impact forces of three structural composite materials without filling. e) Peak impact forces of three structural composite materials filled with Gel. f) Peak impact forces of three structural composite materials filled with PU foam. g) Peak impact forces of the S‐shaped spider web structure filled with different materials. h) Peak impact forces of the spider web structure filled with different materials. i) Peak impact forces of the contrast structure filled with different materials. j) Cushioning efficiencies of the bionic materials at an impact velocity of 0.93 m s^−1^. k) Cushioning efficiencies of the bionic materials at an impact velocity of 2 m s^−1^. l) Comparison of the peak impact forces of six cushioning materials and the integrated bionic composite at two different impact velocities.

A drop hammer test rig was used to perform cushioning tests on the material, as shown in **Figure** **6**a. The test bench was equipped with a 2.5 kg hammer, 10 000 N pressure sensors (Shanghai Li heng Sensor Technology Co., Ltd., China), an NI data acquisition card with 20 kHz acquisition frequency to collect the impact force and NI DAQ Express software to record the impact force‒time data, as shown in Figure 6b,c. In this study, the cushioning performance was characterized by the peak impact force recorded by the force sensor, and a smaller peak impact force indicated superior cushioning performance of the material.^[^ ^9^, ^40^, ^41^ ^]^ The cushioning test was performed at two different impact velocities: 0.93 and 2 m s^−1^.^[^ ^9^, ^42^ ^]^ The test results at both impact velocities showed the same trend: the bioinspired material filled with PU foam had the smallest peak force for the same web structure and different fillings inside the web cavity, as shown in Figure 6g–i, which indicated that PU foam that imitated the structure of pomelo peel could improve the cushioning properties of the material. When the filling inside the web cavity was the same, the structural material with the S‐spider web had the smallest peak force, regardless of whether the cavity was filled with Gel, PU foam or no filling, as shown in Figure 6d–f. When the PU foam and S‐spider web were integrated to form the S‐spider web‐foam, it had the lowest peak force, as shown in Figure 6f, which indicated that the S‐spider web‐foam integrated bionic material had the best cushioning properties. We used the unfilled contrast structure as a reference and calculated the cushioning efficiency of other structural bionic materials. The cushioning efficiency calculation method was based on the literature.^[^ ^43^ ^]^ The S‐spider web‐foam had the highest cushioning efficiency at two different impact velocities, 78% at an impact velocity of 0.93 m s^−1^ and up to 82% at an impact velocity of 2 m s^−1^, as shown in Figure 6j,k.

The cushioning performance of the integrated bionic material S‐spider web‐foam was compared with that of six other cushioning materials, as shown in the Supporting Information, Figure 4. The cushioning test was divided into two different impact velocities, and the test results are shown in Figure 6l. Compared with the other cushioning materials at two different impact velocities, the integrated bionic material S‐spider web‐foam had the smallest peak impact force, and it could reduce the peak impact forces by a factor of 3.5 times compared to silicone foam. **Table** **1** quantifies the cushioning properties of current reported flexible materials. The comparison results showed the bioinspired S‐spider web‐foam material developed in this study exhibits advantages over reported flexible materials regarding cushion performance.

**Table 1:** Comparison of cushioning performance among different materials.

Cushioning performance/Cushioning material	Cushioning efficiency (%)	Reduction of impact force (%)	Control	References/Manufacturer
gel	–	25	Bare gel	[44]
Ionic elastomers	–	36	Blank sample	[45]
non‐Newtonian fluids with PU sponges	–	200	PU sponges	[21]
Liquid crystal elastomer	–	260	Non‐LCE foams	[26]
PUCOIM composites	34	–	PBDMS matrix	[43]
Ecoflex GEL	–	106	Silicone foam	Smoothon, USA
PU Foam	–	122	Silicone foam	Beijing Haibei Si Technology Co.
Silicone (0A)	–	119	Silicone foam	Shin Bon, China
EPE Foam	–	151	Silicone foam	Hangzhou Huafei Packaging Material Co.
EPDM Foam	–	225	Silicone foam	
Bioinspired S‐spider web‐foam (This work)	82	350	Silicone foam	

### 2.4 Theoretical Analysis of Cushioning Performance

During the fall of the hammer, gravitational potential energy was converted into kinetic energy while energy losses were ignored.^[^ ^46^, ^47^ ^]^ When the hammer touched the bionic material, all of its kinetic energy was assumed to be fully absorbed. The energy absorbed by the material was:

(1) $$W=\frac{1}{2}\;m\;v^{2}=\int F\left(x\right)d_{x}$$

Here, “*m*” is the mass of the drop hammer, “*v*” is the impact velocity of the drop hammer, “*x*” and “*F*”are the deformation and compression load of the bionic material, respectively.

**Figure 7:**
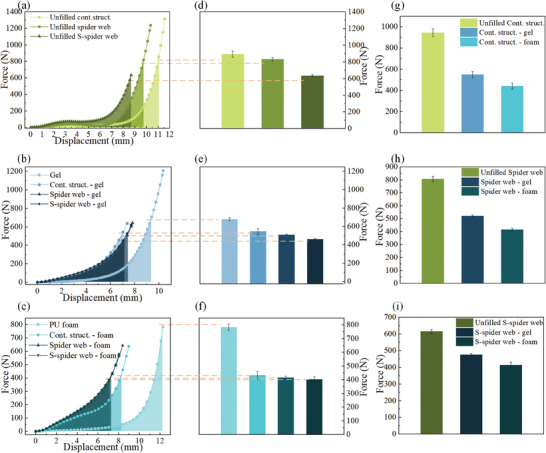
Results of the theoretical analysis of the cushioning performance of bioinspired structural composite materials. a) Load‒displacement curves of three structural composite materials without filling under compressive load; the integrated area below each curve was 1065 N mm in every case. b) Load‒displacement curves of three structural composite materials with gel filling. c) Load‒displacement curves of three structural composite materials filled with PU foam. d) Loads of three structural composite materials without filling in Figure 6a when absorbing the same impact energy. e) Loads of three structural composite materials filled with Gel in Figure 6b when absorbing the same impact energy. f) Loads of three structural composite materials filled with PU foam in Figure 6c when absorbing the same impact energy. g) Loads of comparative structures filled with different materials when absorbing the same impact energy. h) Loads of spider web structures filled with different materials when absorbing the same impact energy. i) Loads of S‐shaped spider web structures filled with different materials when absorbing the same impact energy.

The impact energy was calculated to be 1065 N mm when the impact velocity was 0.93 m s^−1^. Nine bioinspired structural composite materials, Gel and PU foam were tested in static compression tests. The 11 curves obtained from the static compression tests were integrated so that each curve formed a closed graph with the horizontal coordinate with an area of 1065 N mm. That is, each material absorbed the same amount of energy, as shown in **Figure** **7**a–c. For the same energy absorption and the same filling in the web cavity, the lowest load was observed for the structure material with the S‐spider web, regardless of whether the cavity was filled with Gel, PU foam or no filling, as shown in Figure 7d–f. This indicated that the S‐spider web structure is an ideal energy‐absorbing structure, which might be due to the difficulty of bending deformation when the structure is subjected to compressive loading, as proved by the FE simulations, as shown in Figure 5a–c. For the same web structure with different fillings inside the web cavity, the smallest load was observed for bioinspired structural composite materials filled with PU foam, as shown in Figures 7g–i. This indicated that the porous foam structure of the imitated pomelo peel could improve the cushioning properties of the bioinspired structural composite material. When PU porous foam was integrated with the S‐spider web structure to form the integrated bionic material, S‐spider web‐foam, the smallest peak impact force was obtained. This suggests that the S‐spider web structure and the porous foam are coupled and work together to achieve good energy absorption when subjected to impact or compression loads.

### 2.5 Application of Integrated Bionic Materials

**Figure 8:**
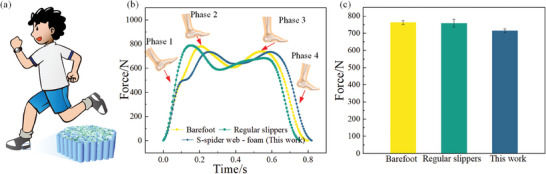
Application demonstration of integrated bionic structural composite materials. a) Bioinspired structural material was embedded into shoe heel and worn by a healthy subject for a walking test. b) Gait measured while wearing different shoes at normal walking velocity. c) Heel impact force while the subject wearing different shoes.

When humans walk, the heel is the first biological tissue to touch the ground, and the heel often generates a large impact force at the moment of heel strike. The peak load on the heel during running can reach 2–3 times the body weight.^[^ ^48^ ^]^ To reduce the impact on the body, insoles are manufactured using materials with excellent cushioning properties.^[^ ^49^ ^]^ To show the potential application of the integrated bionic material in this study in the field of human foot protection, we embedded the integrated bionic material into the heel of a slipper, and performed a walking test, as shown in **Figure** **8**a. The weight of the subject was 75 kg, and the impact force was smallest when wearing bionic insoles compared to walking barefoot and wearing conventional insoles when walking at normal velocity, as shown in Figure 8b,c. That is, the bioinspired structural composite material developed in this study had the best cushioning properties. The force in Figure 8c corresponds to gait phase 2 in Figure 8b, where the heel pad is fully compressed and the heel is subjected to the highest vertical load, and the cushioning material is most needed to reduce the impact force and protect the heel. Therefore, the developed integrated bionic material is expected to be an ideal candidate for bionic insoles and heels of shoes for foot safety and comfort.

**Figure 9:**
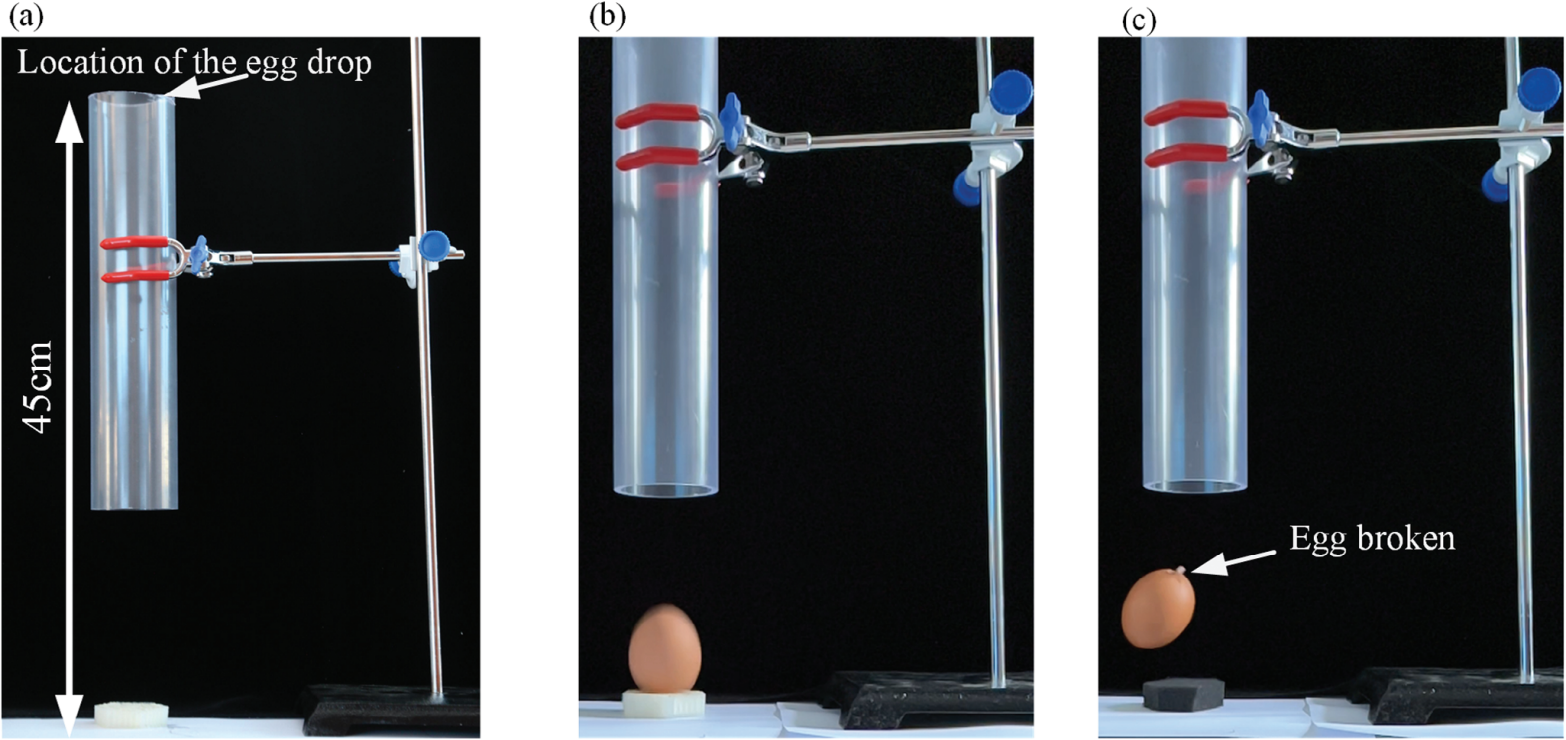
Drop test of an egg. a) Test bench. b) Egg dropped on S‐spider web‐foam. c) Egg dropped on silicone foam.

Fragile products are most prone to damage under impact. We built the egg drop test bench as shown in **Figure** **9**a, more details of the test are in the Supporting Information. Through egg drop tests, this paper assessed the performance of the integrated bionic materials for the protection of fragile products, as shown in the Movies S3 and S4 (Supporting Information). When an egg was dropped on the S‐spider web‐foam, the egg was undamaged, while when an egg was dropped on the silicone foam, the egg broke, as shown in Figure 9b,c. Therefore, the integrated bionic material designed in this study was highly effective in protecting fragile products.

## 3 Conclusion

In this study, an integrated bionic strategy was proposed, and a bioinspired structural composite material with highly efficient cushioning performance was designed on the basis of this strategy. The flexible material was a composite of porous materials with an S‐shaped spider web structure, and it was prepared by the molding method. The composite material integrated the S‐shaped structure of cuttlefish bone, the structure of a spider web and the porous structure of pomelo peel. It had very efficient cushioning performance. At the same impact velocity, compared with six materials (silicone foam, PU foam, Gel, silicone (0A), EPDM foam, and EPE foam) with excellent cushioning properties that were previously reported, the integrated bionic material of S‐spider web‐foam had the smallest peak impact force, i.e., the best cushioning performance. In addition, S‐spider web‐foam could reduce peak impact forces by a factor of 3.5 times compared to silicone foam. The integrated bionic material has a wide range of application prospects for the protection of electronic devices, packaging and transport, insoles for sports shoes and material for the protection of industrial equipment.

## 4 Experimental Section

Preparation of bioinspired structural composite materials: the preparation process of S‐spider web‐foam bioinspired structural material is introduced as an example, as shown in Figure 6 of the Supporting Information. First, 3D printer (Ultimaker S5, Netherlands) was used to print a spider web mould with S‐shaped edges, and the material of the mould was polylactic acid (PLA), the mould structure is shown in Figure 6b of the Supporting Information. Spray the surface of the mould with a small amount of release agent (Smoothen, PA, USA). Mix component A and component B of the silicone (Shin Bon, China) in the ratio of 1:1 by volume and stir thoroughly. The stirred silicone was placed in a vacuum pump for defoaming, and the vacuuming time was 3 minutes. The defoamed silicone is then poured into the mould as shown in Figure 6b of the Supporting Information. The silicone can be fully cured after 4 h at room temperature and the mould is removed, therefore a spider web structure with S‐shaped edges can be prepared, as shown in Figure 6c of the Supporting Information. PU foam (Beijing Haibei Si Technology Co., China) consists of A component and B component mixed according to the volume ratio of 5:2, fully mixed and poured into the web cavity of silicone, as shown in Figure 6c of the Supporting Information. After 30 min, the PU foam was completely cured, and the preparation of S‐spider web‐foam bioinspired structural material was completed.

Cushioning Performance Test: The load sensor is installed under the hammer and fixed. The cushioning material was placed on the upper surface of the force sensor (Shanghai Li heng Sensor Technology Co., Ltd., China), and the slide equipped with the hammer is adjusted to a height of 20 cm (this height corresponds to an impact velocity of 2 m s^−1^) from the surface of the cushioning material. Then the slide was released, and the hammer dropped freely along the slide. The first peak load at impact was recorded using a NI data acquisition card (National Instruments, Inc. USA), and the test was repeated 5 times. The whole experimental process was shown in Movie S1 (Supporting Information).

FE Simulation: Abaqus/standard was used for all FE simulations conducted in the study. Details on the model construction, materials definition, mesh, loading and boundary conditions can be found in Supporting Information.

## Conflict of Interest

The authors declare no conflict of interest.

## Author Contributions

Z.Q.Z. performed investigation, conceptualization, wrote the original draft, and operating tests. Z.H.Q. performed conceptualization, wrote and edited, supervised, funding acquisition. X.W. performed FE simulation and edited. X.L.X. performed FE simulation. B.Y.C. and G.S.S. wrote and edited. X.Y.L. performed investigation, wrote and edited. L.R. performed supervision, methodology, and funding acquisition. L.Q.R. performed supervision.

## Supporting information

Supporting Information

Supplemental Movie 1

Supplemental Movie 2

Supplemental Movie 3

Supplemental Movie 4

## Data Availability

The data that support the findings of this study are available from the corresponding author upon reasonable request.
