# Supplementary material for: Bioinspired Structural Composite Flexible Material with High Cushion Performance
Source: Adv Sci (Weinh). 2023 Dec 3;11(5):2304947. doi: 10.1002/advs.202304947 (PMC10837376; doi:10.1002/advs.202304947)
Supplement: Supplementary file 1 — Supporting Information [file ADVS-11-2304947-s005.pdf]

ADVANCED SCIENCE
Open Access

Supporting Information

for *Adv. Sci.*, DOI 10.1002/advs.202304947

Bioinspired Structural Composite Flexible Material with High Cushion Performance

Zhiqiang Zhuang, Zhihui Qian, Xu Wang, Xiaolin Xu, Boya Chen, Guangsheng Song, Xiangyu Liu*, Lei Ren* and Luquan Ren*

Supporting Information

Bioinspired Structural Composite Material with High Cushion Performance

Zhiqiang Zhuang, Zhihui Qian, Xu Wang, Xiaolin Xu, Boya Chen, Guangsheng Song, Xiangyu Liu*, Lei Ren*, Luquan Ren*

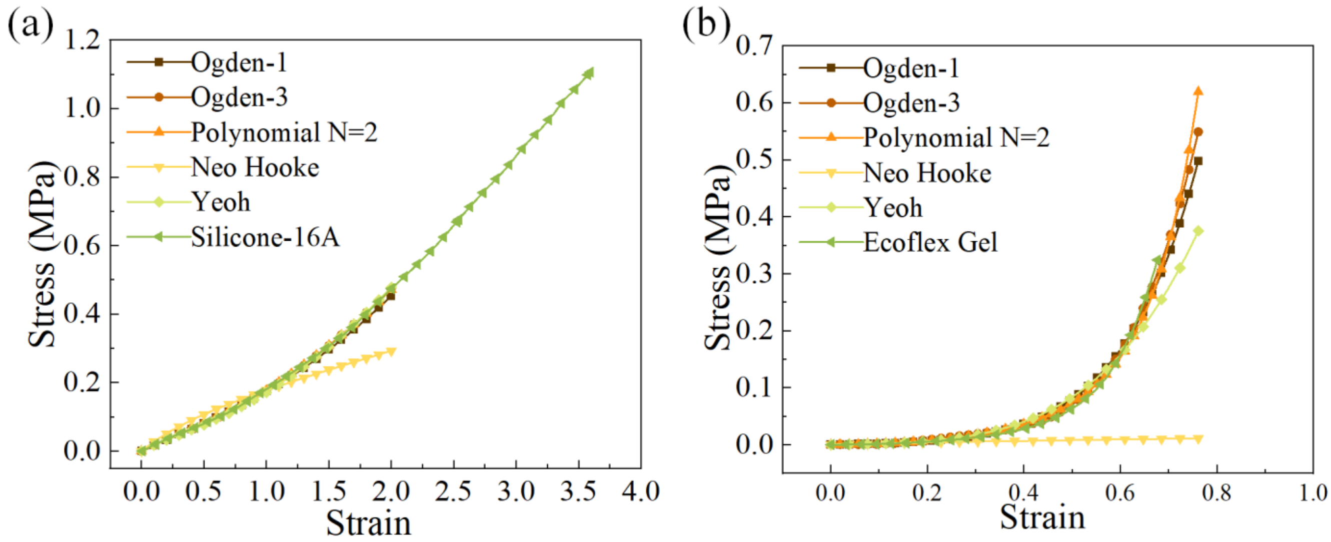

Figure 1.(a) Fitting results for several hyper-elasticity mathematical models for Silicon. (b) Fitting results for several hyper-elasticity mathematical models for Gel.

As can be seen from Figure 1.a), all four hyper-elastic mathematical models, except Neo Hooke, fit the stress-strain curve of silicone very well. As can be seen from Figure 1.b), all three hyper-elastic mathematical models, except Neo Hooke and Yeoh, fit the stress-strain curve of Gel very well. And the parameters of the well-fitted hyper-elastic mathematical models can be used for FE simulation.

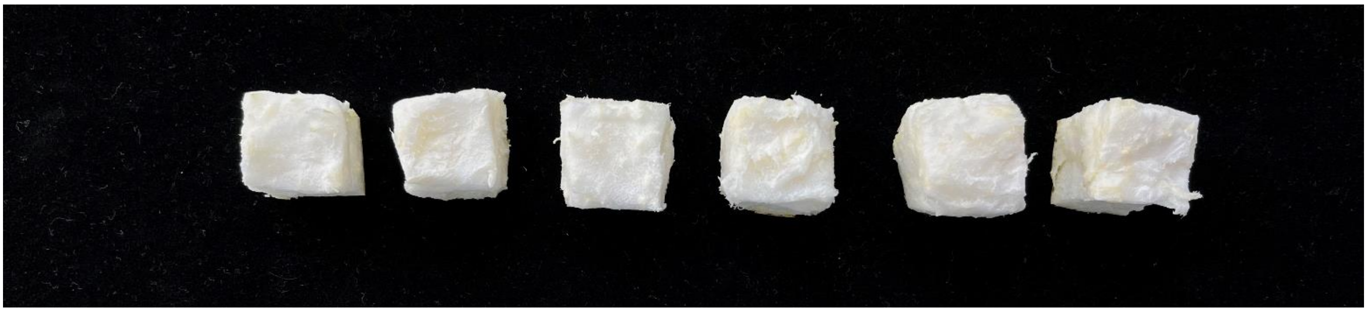

Figure 2. Fresh pomelo peel used for structural observation and experimental studies. Length, width and height are 10mm, 10mm and 15mm respectively.

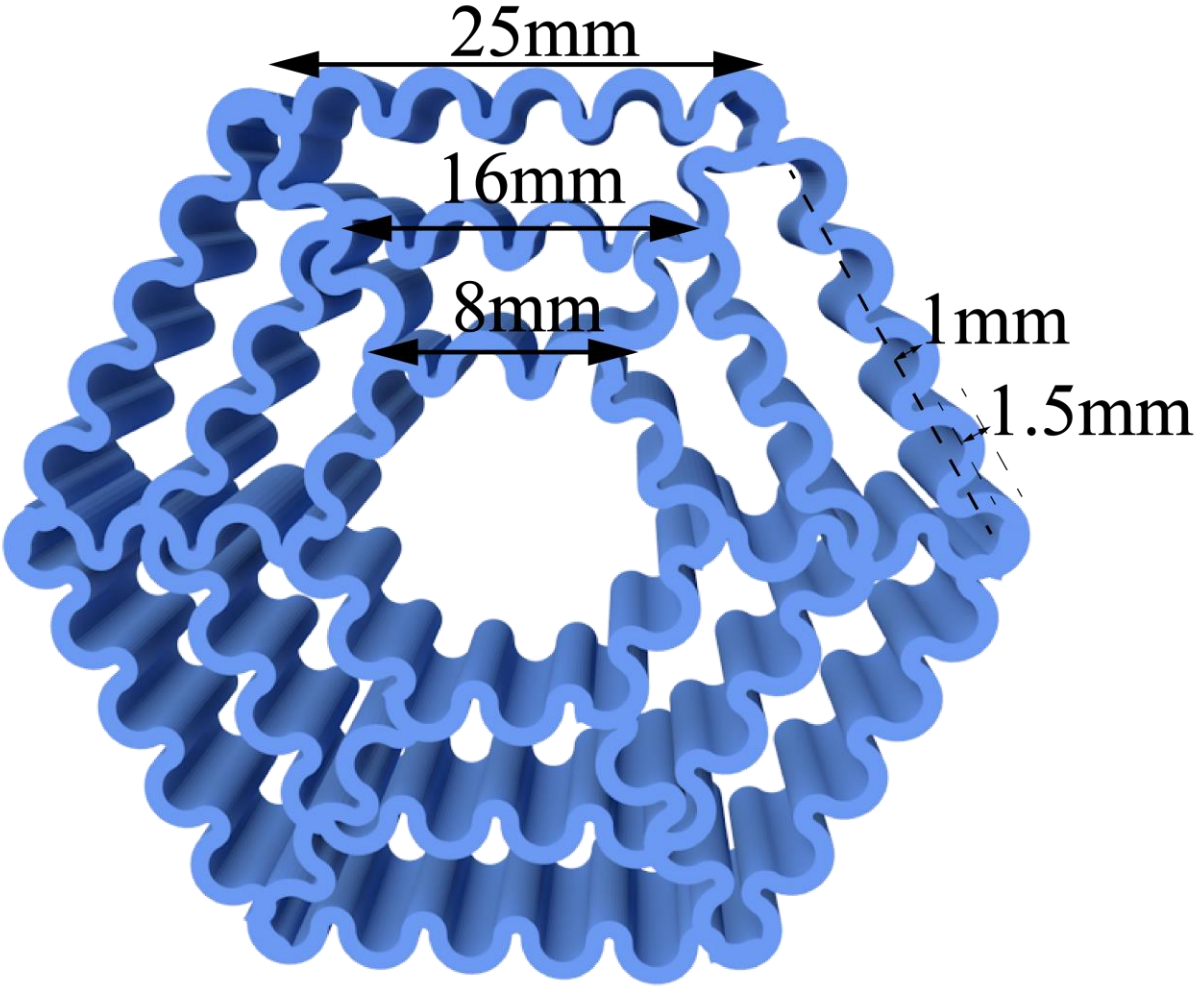

Figure 3. The detailed dimensional information of the S-edge.

Abbreviations	Full name
Unfilled cont. struct.	unfilled contrast structure
Unfilled spider web	unfilled spider web structure
Unfilled S-spider web	unfilled s-type spider web structure
Cont. struct. - gel	contrast structure filled with Ecoflex Gel
Spider web - gel	spider web structure filled with Ecoflex Gel
S-spider web - gel	S-type spider web structure filled with Ecoflex Gel
Cont. struct. - foam	contrast structure filled with PU foam
Spider web - foam	spider web structure filled with PU foam
S-spider web - foam	S-type spider web structure filled with PU foam

Table 1. Abbreviations of the names of bioinspired structural composite materials, with corresponding full names.

	Silicone-16A	Ecoflex Gel	PU foam
**Ogden-1**	$\mu_1 = 6.04 \times 10^{-2}$<br>$\alpha_1 = 3.29$	$\mu_1 = 4.87 \times 10^{-3}$<br>$\alpha_1 = 12.39$	$\mu_1 = 4.59 \times 10^{-2}$<br>$\alpha_1 = 3.39$
**Ogden-3**	$\mu_1 = 0.10, \alpha_1 = -7.18$<br>$\mu_2 = 7.91 \times 10^{-2}$<br>$\alpha_2 = 9.517$<br>$\mu_3 = -0.15,$<br>$\alpha_3 = -19.03$	$\mu_1 = 0.13, \alpha_1 = 6.16$<br>$\mu_2 = 2.44 \times 10^{-5}$<br>$\alpha_2 = 22.48$<br>$\mu_3 = -0.13$<br>$\alpha_3 = 5.527$	$\mu_1 = -0.31, \alpha_1 = 5.82$<br>$\mu_2 = 0.168$<br>$\alpha_2 = 7.03$<br>$\mu_3 = 0.21$<br>$\alpha_3 = 2.65$
**Polynomial N=2**	$C_{10} = 5.64 \times 10^{-2}$<br>$C_{01} = -2.7 \times 10^{-2}$<br>$C_{20} = -2.85 \times 10^{-3}$<br>$C_{11} = 2.51 \times 10^{-2}$<br>$C_{02} = -2.99 \times 10^{-2}$<br>$D_1 = D_2 = 0$	$C_{10} = -0.302$<br>$C_{01} = 0.313$<br>$C_{20} = 2.35$<br>$C_{11} = -6.417$<br>$C_{02} = 4.593$<br>$D_1 = D_2 = 0$	$C_{10} = -0.302$<br>$C_{01} = 0.313$<br>$C_{20} = 2.35$<br>$C_{11} = -6.417$<br>$C_{02} = 4.593$<br>$D_1 = D_2 = 0$
**Neo Hooke**	$C_{10} = 5.06 \times 10^{-2}$<br>$C_{01} = 0$<br>$D_1 = 0$	$C_{10} = 3.84 \times 10^{-3}$<br>$C_{01} = 0$<br>$D_1 = 0$	$C_{10} = 2.75 \times 10^{-2}$<br>$C_{01} = 0$<br>$D_1 = 0$
**Yeoh**	$C_{10} = 3.09 \times 10^{-2}$<br>$C_{01} = 0$<br>$C_{20} = 4.79 \times 10^{-3}$<br>$C_{02} = 0$<br>$C_{30} = -9.0 \times 10^{-5}$<br>$C_{21} = 0$<br>$D_1 = D_2 = D_3 = 0$	$C_{10} = 2.42 \times 10^{-3}$<br>$C_{01} = 0$<br>$C_{20} = 1.43 \times 10^{-2}$<br>$C_{02} = 0$<br>$C_{30} = 2.00 \times 10^{-2}$<br>$C_{21} = 0$<br>$D_1 = D_2 = D_3 = 0$	$C_{10} = 3.10 \times 10^{-2}$<br>$C_{01} = 0$<br>$C_{20} = -2.07 \times 10^{-2}$<br>$C_{02} = 0$<br>$C_{30} = 1.73 \times 10^{-2}$<br>$C_{21} = 0$<br>$D_1 = D_2 = D_3 = 0$

Table 2. Mathematical model parameters of hyper-elasticity of silicone, Gel and PU foam.

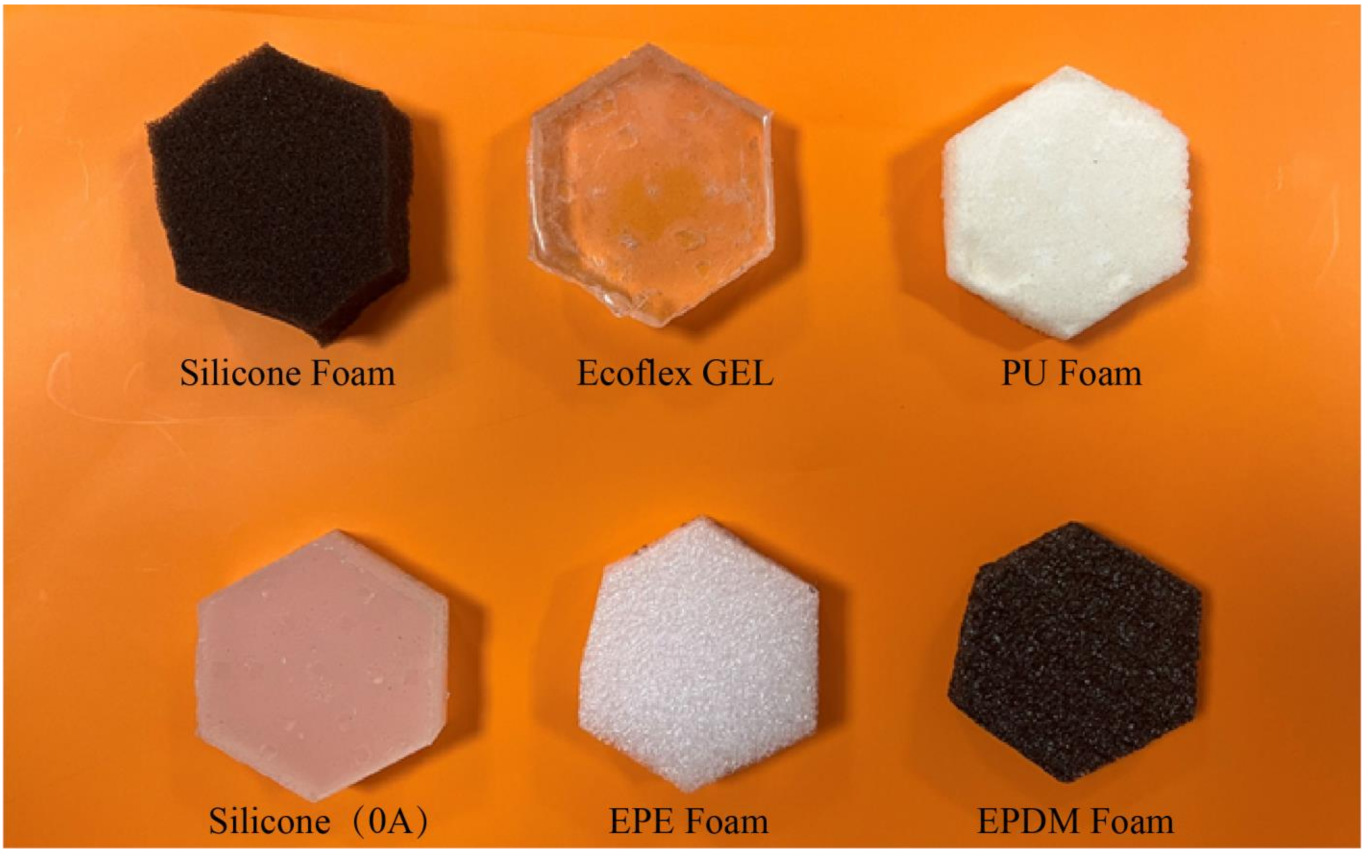

Figure 4. Six materials with excellent cushioning properties, all of the same structural dimensions.

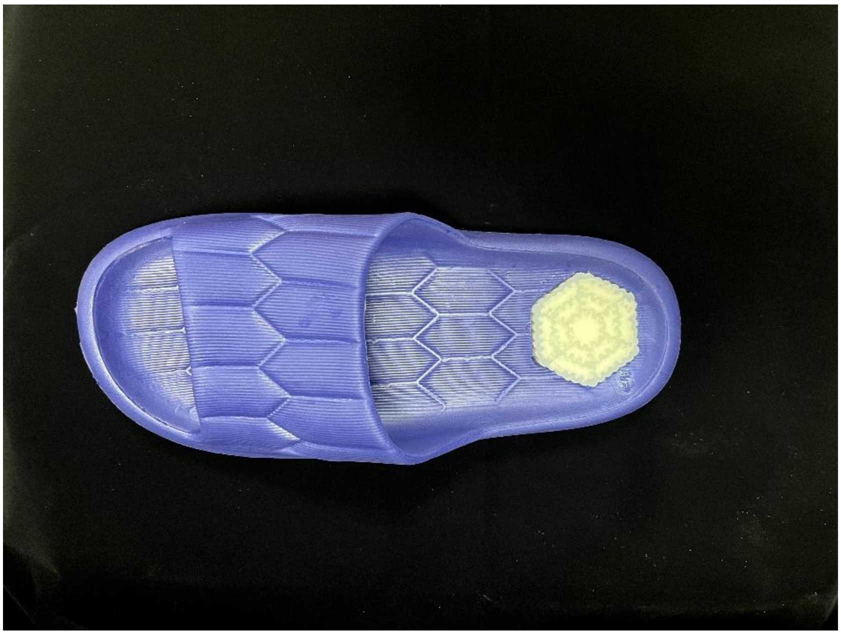

Figure 5. The bioinspired structural material being embedded into the heel of the shoe.

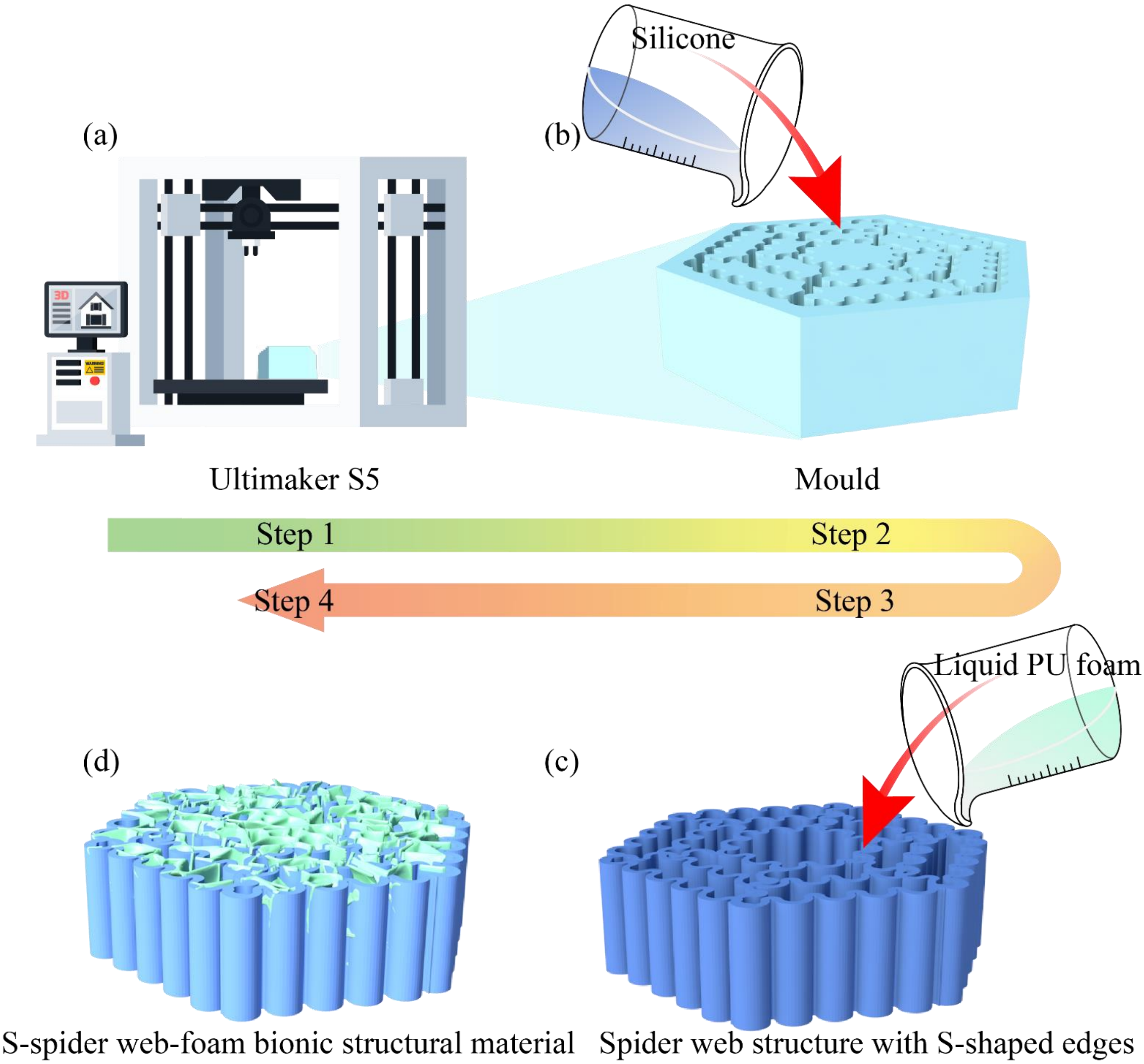

Figure 6. Preparation process of bioinspired structural material. a) Schematic of 3D printer. b) Defoamed silicone is poured into the mould. c) Liquid PU foam is poured into the web cavity of silicone. d) Schematic of S-spider web-foam bionic structural material.

Egg drop test

In this study, an egg drop test setup was also constructed with the keeper clamping the guide cylinder. The guide cylinder is used to ensure the egg falls on the bionic material when it falls freely. The overall height of the test setup is 45 cm. The egg with mass of 60 g was placed at the top of the guide cylinder and was released freely. The video of the egg drop was recorded by using a high speed camera. The cushioning performance of the material was evaluated in terms of whether the egg was broken or not. The test results showed that the egg did not break when it fell on the S-spider web-foam, while it broke when fell on the silicone foam. This also shows that the developed S-spider web-foam has a good cushioning effect and may have good potential in packaging protection of fragile products.

Abaqus (Simulia, Providence, USA) is used to perform FE simulation of the bioinspired structural composite materials. The FE simulation consists of four components, which are the S-shaped spider web structure, the bionic porous material of pomelo peel that fills the cavity, and two rigid bodies, as shown in Figure 7 in supporting material. Bionic porous material of pomelo peel was defined as Yeoh hyper-elastic material, S-shaped spider web structure was defined as Ogden-3 hyper-elastic material. The specific material parameters were shown in Table 2 in the supporting material. The mesh type of bionic porous material of pomelo peel is 3D hexahedron with a size of 1.4 mm. The mesh elements of S-shaped spider web structure is 3D tetrahedron and has a mesh size of 2 mm. The contact between the upper and lower surfaces of bioinspired structural material and rigid body is defined as a frictionless surface-to- surface contact, as shown in Figure 7 in Supporting material. Tie constraints were used between bionic porous material of pomelo peel and S-shaped spider web structure. Abaqus/standard solver was employed to conduct a quasi-static simulation. The compressive displacement was defined as 1.2 mm, to result in 10% compressive strain of the model.

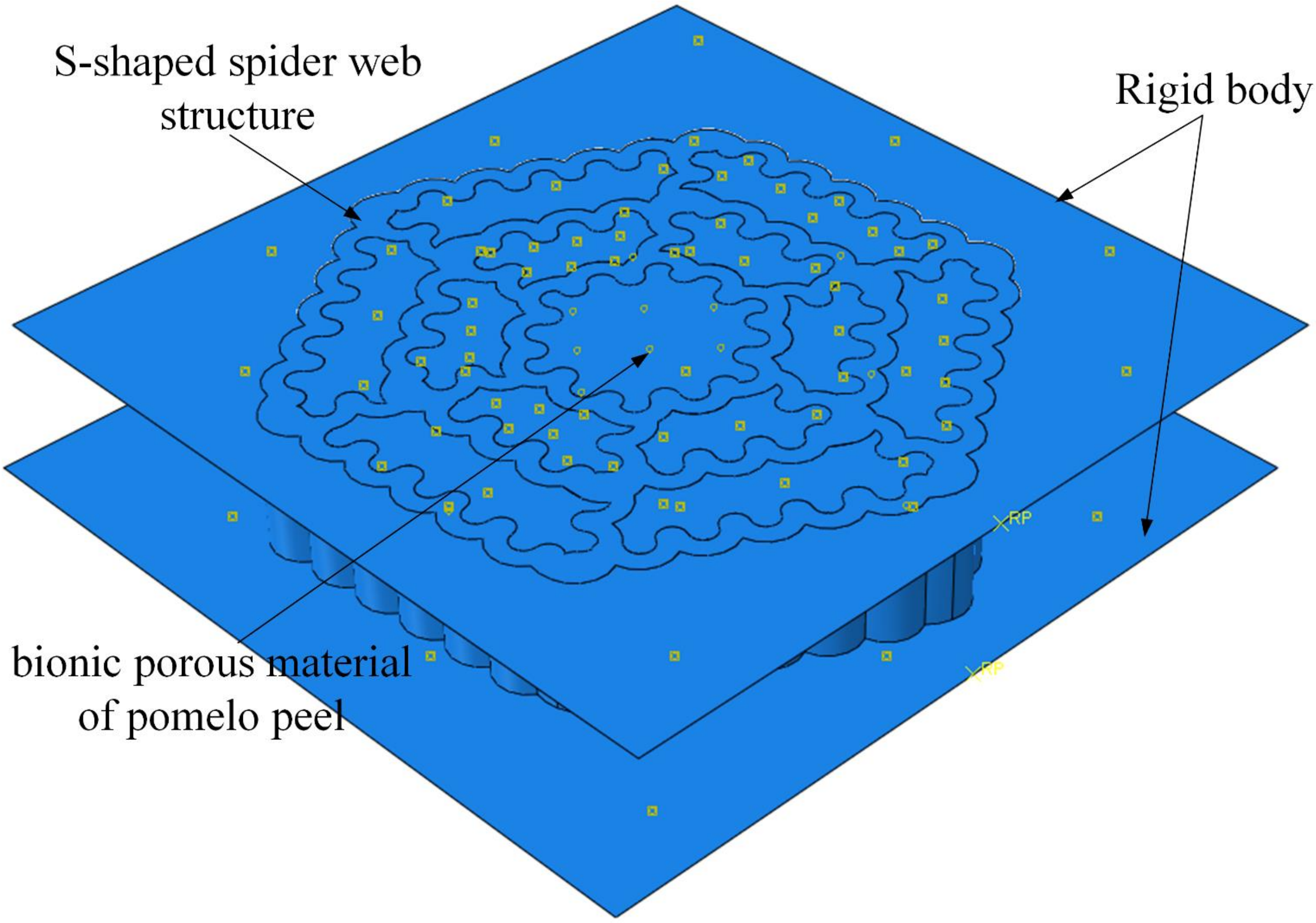

Figure 7. Models for FE simulation.
